# Research on the construction strategy of China’s rural emergency linkage system from the perspective of systems theory—based on the survey of six regions in China

**DOI:** 10.3389/fpubh.2025.1611273

**Published:** 2025-07-04

**Authors:** Xinyang Huang, Yuning Jiao, Zihao Deng, Tianyao Wang, Yuyao Hu, Yuting Liu, Wei Wang, Wei Nie

**Affiliations:** ^1^Beijing Shijitan Hospital, Capital Medical University, Beijing, China; ^2^Beijing Anzhen Hospital, Capital Medical University, Beijing, China; ^3^School of Public Health, Capital Medical University, Beijing, China; ^4^School of Basic Medicine, Capital Medical University, Beijing, China

**Keywords:** rural China, pre-hospital emergency care, coordination, systems theory, construction strategy

## Abstract

**Purpose:**

This study aims to understand the major challenges facing China’s rural pre-hospital emergency care in terms of coordination and cooperation. We focus particularly on the three-tier emergency care providers and their surrounding support units to explore the systematic and strategic gaps within the system. Based on these findings and general systems theory, we propose construction strategies for China’s rural emergency linkage system. This research provides new approaches for improving China’s overall rural emergency care capacity and offers reference points for rural emergency care development in other countries.

**Methods:**

We visited 6 rural areas across 5 provinces and municipalities in China (Beijing, Shandong, Jiangsu, Zhejiang, and Jiangxi). We conducted semi-structured interviews with 28 grassroots emergency care personnel, including villagers with emergency care experience, village doctors, village officials, and emergency center staff. Through these interviews, we conducted in-depth research on the challenges facing rural emergency care coordination and gathered specific recommendations for improvement.

**Results:**

In current rural pre-hospital emergency care practice in China, coordination between different units remains insufficient, and the overall system development is still incomplete. The three-tier emergency care providers lack adequate information sharing, clear division of emergency responsibilities, and personnel exchange. Additionally, social and environmental factors such as funding limitations, compensation distribution issues, and rural transportation planning create barriers to rural emergency care development.

**Conclusion:**

The construction strategy for China’s rural emergency linkage system consists of two main components: the “emergency central system” and the “emergency peripheral system.” The central system focuses on integrating information sharing, medical care coordination, and human resources among emergency care providers. The peripheral system addresses economic support, road and address management, and strengthening emergency transportation capacity. Together, these systems can effectively enhance coordination within the emergency care system and improve the overall effectiveness of China’s rural emergency care.

## 1 Introduction

The Chinese Communist Party has repeatedly emphasized the importance of comprehensively advancing the rural revitalization strategy and building modern, beautiful countryside (1). Rural pre-hospital emergency care system development represents an extremely important and critical component of this rural revitalization effort (2). Pre-hospital emergency care refers to emergency treatment provided to critically ill patients outside of hospitals (3). The pre-hospital emergency care system is a complex medical system that covers the entire process before, during, and after pre-hospital emergency care, including many aspects such as emergency resource allocation, emergency personnel supply, and emergency operation mechanisms.

Currently, most rural areas in China have incomplete pre-hospital emergency care systems, with inadequate three-tier main providers, insufficient coordination, and lagging overall effectiveness (4). Taking acute myocardial infarction as an example, approximately 20% of patients in China’s urban areas can reach hospitals for emergency treatment via ambulance, but in rural areas this proportion is less than 10% (5, 6). Against this background, our study investigates rural areas across 6 regions in 5 provinces and municipalities in China. We analyze the challenges that various rural emergency care providers face in system construction and coordination. Using general systems theory as our theoretical foundation, we make judgments about optimization goals for different emergency care providers and construct an emergency linkage system suitable for China’s rural areas. Our aim is to optimize coordination between different units and strengthen emergency care capacity, providing reference points for systematic improvement of rural emergency care systems in China and other countries.

## 2 Materials and methods

### 2.1 Study participants

Between November 2022 and December 2024, our research team visited 6 rural areas across 5 provinces and municipalities in China: M District and T District in Beijing, L City in Shandong Province, W City in Jiangsu Province, T City in Zhejiang Province, and R City in Jiangxi Province. These six locations provide good representation in terms of geographical distribution, economic development, and natural landscape features. From a regional distribution perspective, the selected areas cover different provinces from north to south, taking into account regional differences between northern and southern China. Additionally, the selected regions have varying Gross Domestic Product(GDP) levels, which helps reflect both commonalities and differences in rural emergency care systems under different economic backgrounds. For example, Beijing had the highest GDP in 2023 (approximately 4.38 trillion yuan), followed by W City and T City, while L City and R City had relatively lower economic development levels (7). In terms of geographical features, the study areas include plains, hills, mountains, and basins, which helps provide a comprehensive analysis of rural emergency care practices under diverse geographical conditions.

Our team used purposive sampling to select a total of 28 participants, including rural residents with emergency care experience, village doctors, village officials, and emergency center staff from each location. We conducted in-depth interviews focusing on the current problems and recommended solutions for rural emergency care. The basic information of interview participants is shown in Table 1. We determined sample size using the information saturation principle: when organizing interview materials revealed no new themes emerging, we considered information saturation to be reached.

**Table 1:** General information of interviewees.

No.	Personnel	Region	No.	Personnel	Region
S1	Villager	JiangSuW	S15	Village doctor	ShanDongL
S2	Villager	JiangXiR	S16	Village doctor	ShanDongL
S3	Villager	JiangXiR	S17	Village doctor	BeiJingM
S4	Villager	ShanDongL	S18	Village doctor	ZheJiangT
S5	Villager	ShanDongL	S19	Emergency doctor	ZheJiangT
S6	Villager	BeiJingM	S20	Village official	JiangSuW
S7	Villager	BeiJingT	S21	Village official	JiangXiR
S8	Villager	ZheJiangT	S22	Village official	BeiJingM
S9	Villager	ZheJiangT	S23	Village official	ZheJiangT
S10	Villager	BeiJingT	S24	Village official	BeiJingM
S11	Village doctor	JiangSuW	S25	Emergency center staff	JiangXiR
S12	Village doctor	JiangXiR	S26	Emergency center staff	JiangSuW
S13	Village doctor	JiangXiR	S27	Emergency center staff	BeiJingM
S14	Village doctor	ShanDongL	S28	Emergency center staff	ZheJiangT

### 2.2 Study methods

This study employed semi-structured interviews, collecting data through face-to-face in-depth interviews or written questionnaires. We chose this method because it can more effectively capture the essential problems of China’s rural emergency care, which represents a complex medical practice. The advantage of qualitative research lies in its ability to obtain rich experiences, genuine feelings, and behavioral logic in specific situations from key stakeholders (villagers, village doctors, village officials, and emergency center staff) through in-depth, open dialogue using authentic, vivid narratives from interview participants. This approach helps reveal deep mechanistic issues such as “why coordination between the three-tier emergency care providers is poor” and “how social and environmental factors constrain emergency care development.” Semi-structured interviews allow researchers to ask follow-up questions flexibly and explore unexpected viewpoints and localized insights from interview participants. This provides comprehensive, detailed descriptions of the real challenges and inherent complexity of rural emergency care, establishing a solid, practice-based evidence foundation for subsequent in-depth analysis of causes and optimization model development. This type of deep understanding is difficult to obtain through quantitative methods such as questionnaires.

Before conducting interviews, research team members had already reviewed relevant literature, developed and familiarized themselves with the interview guide, and thoroughly understood the interview purpose, content, and procedures. They also learned certain interview techniques to ensure smooth interview processes. The interview guide was developed by research team members through literature review and current information gathering, formed after three rounds of discussion and revision, focusing closely on core points such as the current status, problems, and optimization strategies for rural pre-hospital emergency care. Interview guides for villagers, village doctors, and village officials can be found in our published work “Grassroots Extension of Rural Pre-hospital Emergency Care: Building ‘Villager-Village Doctor’ Self-rescue and Mutual Rescue Links” (5). The interview guide for emergency center staff included the following: ① Basic ambulance data: ambulance distribution and coverage areas; emergency call frequency; response times; rescue success rates (empty runs, return trips, etc.); ② Emergency station distribution and local emergency care models; ③ Current pre-hospital emergency call systems: Are calls unified? How common is it for patients to call hospitals directly? Is information about rural emergency patients uniformly reported upward? ④ Current emergency system role in emergency personnel training: What are the shortcomings in this area? ⑤ Current emergency care fees: Are there fee-related disputes? Are there incentive policies for medical staff participating in emergency care? ⑥ What do you think are the biggest shortcomings in current rural pre-hospital emergency care? What do you think would be the most effective solutions?

Research team members first introduced the study purpose and process to participants, asked them to sign informed consent forms, and with their permission, recorded interview content and preserved written responses. During interviews, research team members carefully recorded participants’ conversation content and emotional changes. Each interview lasted 10–20 min, with some in-depth interviews extending to 30–40 min. After interviews, research team members thanked participants and promised that interview content would only appear anonymously in social science research.

After interviews, research team members transcribed interview recordings word by word and analyzed the data following these steps: ① Carefully read all original materials and collected meaningful statements; ② Summarized repeatedly appearing viewpoints; ③ Gathered, categorized, and refined themes from summarized viewpoints; ④ Reflected on participants’ viewpoints and behavioral responses to form initial theme descriptions; ⑤ Integrated organized materials to specify theme content. This process ultimately produced 28 high-quality original interview records totaling over 60,000 Chinese words.

## 3 Results

The current operational efficiency of China’s rural pre-hospital emergency care system is concerning. As S1 mentioned: “When my father had his medical emergency, it took nearly 10 min to get through to 120, and by the time the ambulance arrived, more than half an hour had passed. We had no choice but to take him to the hospital ourselves first.” S6 reflected: “The roads in our village are too narrow for ambulances to get through easily. Sometimes they just stop at the village entrance, and we have to use a flat cart to push the patient out.” These real situations of delayed response, obstructed pathways, and disconnected information demonstrate that China’s rural emergency care faces systematic problems in coordination mechanisms and resource security. Based on our field visits and interview findings, we conducted an in-depth analysis of the current status of coordination among various rural emergency care providers. We have organized the general and widespread problems discovered as follows.

### 3.1 Lack of linkage and communication among the three-tier emergency subjects: village clinics, township health centers, and county/city hospitals

Emergency information sharing networks between the three-tier providers are either non-existent or ineffective. S19 said: “The problem is that when patients are transferred to us, both we and the handover staff do not really understand the patient’s condition. Actually, this information could be clarified through preliminary examination work during emergency care.” Liu Jiamin and colleagues found through research in rural areas of Shiyan City, Hubei Province, that most ambulances lack even the most basic vehicle communication equipment (8). In terms of communication technology, rural pre-hospital emergency care lacks unified information platform management and smart technology integration. Mature information and technology applications have not received adequate attention and development (9). The real-time information sharing systems between the three-tier emergency care providers are incomplete or have not been established at all. County-level hospitals and lower-level medical institutions cannot achieve effective information connection, which leads to problems such as repeated examinations, delayed diagnosis, and delayed preparation of emergency equipment and medications. This ultimately delays patient treatment, and emergency doctors are more likely to make treatment errors when working without adequate preparation.

Division of emergency responsibilities and hierarchical structure between the three-tier providers is unclear. The “Healthy China 2030” Planning Outline states: “Establish clear goals and responsibilities, and develop coordination mechanisms between medical and health institutions of different levels, categories, and ownership structures (10)“. However, most rural areas currently rely heavily on the single force of county-level hospitals for pre-hospital emergency care, failing to form a clear division of labor with step-by-step emergency relay coordination. This is not conducive to efficient emergency processes, and it becomes difficult for doctors to effectively distribute and coordinate tasks (11). As S25 said: “After patients arrive, we often do not understand many aspects of their condition, so we cannot prepare in advance, and the doctors who transported them do not brief us either.” Meanwhile, the widespread mentality among villagers of preferring first visits to higher-level hospitals exists commonly, with low trust in lower-level medical institutions, which becomes especially evident in emergency situations (12).

Insufficient communication and exchange of emergency personnel between the three-tier providers. S14 said: “Now doctors from county and city hospitals rarely come to our area, and there’s very little communication. Basically, everyone just does their own work.” Due to economic and other objective factors, the gap between urban and rural medical levels is enormous (13). Having upper-level hospitals send personnel to guide lower-level hospitals in emergency care guidance and exchange activities has profound significance for overall urban–rural emergency care development. However, our research found that due to lack of specific command and planning by emergency centers and other departments, insufficient communication concepts, plus institutional factors, there is little or extremely limited personnel flow and job rotation between county-level hospitals and lower-level medical institutions. This makes it difficult to achieve inter-institutional medical collaboration. Mobile systems such as emergency care going to rural areas and emergency care going to villages have not yet been established by county-level hospitals, which negatively affects both narrowing urban–rural medical gaps and smooth emergency care processes.

### 3.2 Social environmental factors creating difficulties for rural pre-hospital emergency system development

Insufficient dedicated funding for rural pre-hospital emergency care construction. The issue of inadequate financial support was raised multiple times during interviews. Pre-hospital emergency care has a public welfare nature and belongs to non-profit services, yet government financial support is relatively scarce. The widespread problem of “emphasizing in-hospital care over pre-hospital care” in rural and even urban areas represents an important obstacle affecting emergency care system construction and improvement. Local governments need to be responsible for purchasing emergency medical equipment, recruiting professional emergency personnel, and conducting building renovation and infrastructure construction work. High operational and maintenance costs lead to continuously increasing financial pressure. Additionally, township medical institutions have weak revenue-generating capacity themselves. Developing pre-hospital emergency care requires simultaneously dealing with operational losses and medical dispute risks. This dual challenge makes construction and maintenance of rural emergency care stations even more difficult (14).

Current compensation distribution systems make it difficult to retain grassroots emergency care talent. S25 said: “Pre-hospital emergency personnel work 24-h shifts. With one phone call, we must dispatch within 2 min and cannot leave the work area for 24 h.” S28 said: “I think the pay is still too low, and we are essentially on duty 24 h a day.” Most frontline emergency doctors we interviewed reported low pay and heavy workloads. Emergency care often faces the most challenging and high-pressure rescue situations, requiring high levels of technical skill, experience, and composure from doctors. The hard work of emergency doctors is undeniable, but in many areas, emergency doctor wages are often lower than those of other doctors at the same level, seriously affecting the work enthusiasm of frontline emergency doctors and causing continuous talent loss.

Chaotic and disorganized rural road planning and address numbering systems lead to inefficient emergency location finding. S21 said: “Rural villagers are too scattered, house numbers follow no rules, and small roads are too narrow, making emergency care really inconvenient.” Our field visits found that rural road conditions are complex, house arrangements follow no patterns, and address numbering is chaotic. We learned that disorganized road planning and address numbering is a “chronic problem” in China’s rural areas that has long received insufficient attention. However, in actual pre-hospital emergency care processes, confusing routes and unclear address numbering often create enormous difficulties and time waste for ambulances entering villages to find emergency patients, greatly reducing emergency care success rates. Standardizing road construction, optimizing address numbering systems, and strengthening daily maintenance not only helps improve emergency transportation efficiency but also provides convenience for postal communications, emergency rescue, and other fields.

## 4 Discussion

### 4.1 Current challenges

China’s pre-hospital emergency care system development began in the 1950s. After more than 40 years of development, the country has established emergency care networks centered on major cities such as Beijing, Shanghai, and Wuhan. However, due to factors including large population size and unbalanced regional economic development, China’s pre-hospital emergency care overall shows characteristics of large regional differences, non-unified standards, shortage of pre-hospital emergency personnel, and poor pre-hospital emergency informatization. Among these issues, low pre-hospital emergency care levels are particularly evident in rural areas (15–17). Our study shows that disconnected coordination between different emergency care units represents an important challenge in rural pre-hospital emergency care practice. Under ideal conditions, the three-tier emergency care providers of “village clinics—township hospitals—county-level hospitals” should form a tightly connected emergency care chain. However, the reality is not encouraging. The three-tier providers commonly face problems of high information barriers, unclear division of emergency responsibilities, and lack of personnel exchange. Information cannot be shared in real-time, leading to inadequate treatment preparation, repeated examinations, and delays. Unclear hierarchical division of labor causes unclear responsibilities, chaotic processes, and resource misallocation. The lack of upward and downward personnel flow solidifies and worsens the urban–rural emergency care capacity gap. Furthermore, constraints from social and environmental factors cannot be ignored. Insufficient dedicated funding for pre-hospital emergency care leads to delayed emergency station construction, outdated equipment, and operational difficulties. Weak compensation incentives for grassroots emergency personnel make it difficult to attract and retain professional talent, resulting in poor team stability. Additionally, rural infrastructure has shortcomings, especially disorganized road planning and unclear address numbering, which seriously hinder ambulances’ rapid response and precise location finding. These challenges interweave with each other, together forming systematic obstacles that prevent rural emergency care effectiveness improvement.

Solving these problems urgently requires clarifying core task objectives for various emergency care stakeholders and promoting their coordinated efforts. The main providers of emergency care—the three-tier emergency care providers—have core tasks of clarifying responsibilities at each level and establishing standardized, progressive emergency care and referral processes. They must also break through “village-township-county” information barriers, establish real-time sharing platforms, and ensure seamless flow of patient condition, dispatch, and handover information (15, 16). Indirect providers of emergency care have main objectives of strengthening financial security, optimizing infrastructure support, and actively mobilizing social forces to provide good social environmental support for rural emergency care (18).

Currently in rural China, although governments and medical institutions at all levels have recognized rural emergency care problems and taken some measures, significant gaps remain in systematic integration and coordination mechanism construction. Many efforts often focus on single elements (such as increasing ambulance numbers) or single levels (such as improving county-level hospital capacity) (18, 19), lacking top-level design and coordination mechanisms that view the three-tier providers and their surrounding units as an organic whole. Information platform construction is fragmented and lacks unified standards (20). Inter-level cooperation often relies on temporary arrangements or administrative orders, lacking institutionalized division of labor, collaboration, and incentive mechanisms (5). Integration and utilization of social support factors are also clearly insufficient (21). This “treating symptoms rather than causes” partial optimization approach makes it difficult to fundamentally solve the problem of overall emergency care ineffectiveness caused by systematic disconnection and resource fragmentation.

### 4.2 Systems theory and strategy construction

**Figure 1:**
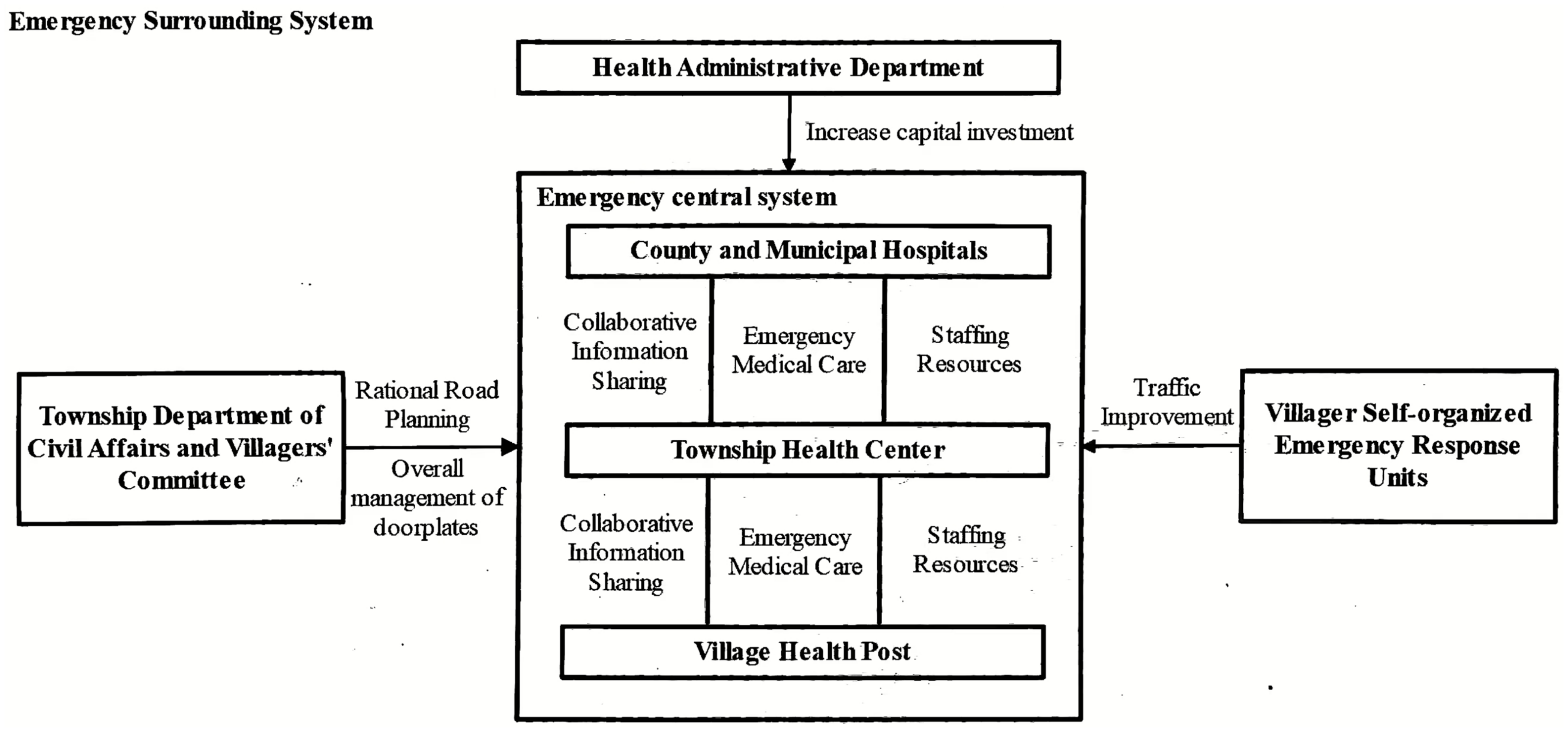
Rural emergency linkage system construction strategy.

We can see that optimizing rural pre-hospital emergency care systems involves coordination of multiple dimensions, multiple stakeholders, and multiple levels of factors. This is essentially a complex systems engineering project. Based on Bertalanffy’s general systems theory (22), our study proposes a rural emergency linkage system construction strategy consisting of an “emergency central system” and “emergency peripheral system” (Figure 1). This strategy follows the systems principle of holism, emphasizing the synergistic effects of multiple emergency care providers working together rather than the development of any single unit, and emphasizes coordination between system components (23). Based on the systems principle of hierarchy (24), our study’s emergency central system is further subdivided into three levels: village clinics, township hospitals, and county-level hospitals. Each level has clearly different emergency care responsibilities, but they are interconnected in information transmission, resource allocation, and other aspects. Additionally, the systems theory principle of openness indicates (25) that rural emergency care systems, as open systems, need dynamic interaction with external environments (such as policy, economic, and transportation factors). Therefore, we also introduce the concept of emergency peripheral systems, covering elements such as financial investment, road and address management, emergency capacity supplementation, and public participation to create an external environment conducive to rural emergency care system operation. The emergency central system and emergency peripheral system support each other and work together, forming dual pillars that ensure rural emergency care effectiveness improvement. The dynamic coordination between these two systems can effectively break through existing rural emergency care information barriers and resource fragmentation problems, providing an operational systematic solution for constructing cross-departmental and cross-regional emergency care coordination mechanisms. Together, they help achieve coordination between emergency care units and overall improvement.

### 4.3 Central emergency system: complementary linkage and close coordination

As the three-tier emergency care providers forming the emergency central system, improving their overall coordination is particularly necessary for strengthening urban–rural emergency care cooperation, breaking down urban–rural emergency care barriers, and narrowing urban–rural emergency care gaps.

Optimize communication equipment and strengthen information sharing. Real-time emergency information is crucial for emergency transportation efficiency, so establishing or improving information sharing channels should be prioritized. Information sharing is mainly reflected in grassroots institution doctors being able to collect patient physical data in a short time and promptly inform upper-level institution emergency doctors, shortening their preparation time and thereby improving rescue success rates. Constructing emergency information sharing channels can fully utilize information technology to achieve orderly allocation and expert guidance, shorten emergency response radius, and strengthen urban–rural emergency care cooperation. For example, on the basis of improving call reception and dispatch systems, connections between pre-hospital emergency care and in-hospital treatment information can be achieved, and regional integrated information platforms can be gradually established (26). As another example, we can explore introducing the American MPDS emergency priority dispatch system to achieve call classification and grading, while implementing electronic management of pre-hospital emergency cases to ensure emergency information chain completeness (27, 28). Furthermore, with the help of digital and intelligent tools such as “smart 120 ambulances” and “remote consultation platforms” (29), real-time uploading of vital signs and remote expert guidance can be achieved while patients are being transported, promoting closed-loop information coordination of “entering hospital upon boarding the ambulance” (30). In terms of infrastructure construction, 5G + Beidou positioning technology can be introduced to achieve intelligent patient location and route planning, improving emergency response accuracy and timeliness (31).

Clarify responsibilities at each level and achieve integrated emergency care processes. Throughout the entire emergency care process, operations implemented by medical providers at each level should have clear division of labor, step-by-step progression, and improved efficiency. Village doctors’ responsibilities mainly involve assessing patient conditions and providing simple treatment (32, 33). During transportation, emergency personnel operations mainly involve monitoring and support, while in-hospital emergency care is key to solving patients’ actual medical problems. Clarifying responsibilities for all parties, having everyone perform their duties, and reducing ineffective operations and wasted time can make the overall emergency care process organized and efficien (34). Based on this foundation, corresponding referral processes and operational standards should be developed according to regional disease patterns and common emergency types to improve process standardization. Additionally, cross-level cooperation mechanisms should be explored, such as having county-level hospitals organize Red Cross volunteer service teams to regularly conduct rural visits and health screenings, achieving early disease screening and treatment at the pre-hospital stage and improving prevention and control levels for sudden illnesses. Meanwhile, relying on county medical communities can promote standardized training and unify emergency record standards, further improving operability and consistency in three-tier coordination.

Strengthen personnel exchange and balance resource allocation. The three-tier emergency care providers should strengthen emergency personnel exchange and communication to break down urban–rural emergency care barriers. Compared to county-level hospitals, medical workers at township hospitals and village clinics generally have lower education levels and insufficient emergency technique mastery, clearly showing incomplete and unbalanced urban–rural medical care. Therefore, upper-level county-level hospitals should actively undertake emergency assistance and guidance for lower-level rural health institutions, conducting long-term mobile job rotation and emergency care village visits. Related compensation and performance systems should also be improved to encourage emergency doctors’ cross-institutional and cross-urban–rural exchange and cooperation, strengthening emergency technology and talent team building at lower-level rural health institutions. The three-tier emergency care providers should coordinate and communicate with each other, improve medical resource allocation, and increase medical resource utilization rates (35). Meanwhile, cross-institutional rotation systems can be established to promote county-level hospital doctors’ regular support at grassroots health institutions and organize emergency care specialists to conduct grassroots teaching tours and operational guidance. Using modern information technology for real-time emergency care command and remote expert consultation can achieve urban–rural medical resource sharing and reasonable medical resource allocation. This will significantly improve rural medical emergency care conditions and gradually form an emergency care model of “major illnesses at major hospitals, minor illnesses at smaller hospitals,” achieving the goal of “minor illnesses at grassroots levels, major illnesses at hospitals, and recovery back to grassroots levels” (36).

### 4.4 Pre-hospital emergency peripheral system: multi-party cooperation and macro-level support

Health administrative departments: Increase rural emergency care funding “Economic foundation determines superstructure” (37). Health administrative departments should fully recognize the shortcomings and urgency of rural emergency care and ensure dedicated emergency development funding so that the optimization measures mentioned above, such as resource allocation and information sharing, can truly be implemented. Additionally, the contradiction between high emergency care costs and low villager income also needs to be gradually resolved through expansion and improvement of rural medical security systems. Including emergency care costs in rural medical insurance coverage, increasing emergency medication reimbursement rates, and government intervention in emergency transportation fee regulation can work together to reduce villagers’ “concerns about calling for help” (38).

Township civil affairs departments and village committees: Reasonable road planning and coordinated address management. Emergency care environment is one of the key factors determining emergency care effectiveness and relates to rural residents’ sense of access and participation in emergency medical care (10). This requires improvement by township civil affairs departments and village committees. Related expenditures for improving village appearance and infrastructure construction should be increased, with reasonable road planning and coordinated address management, plus good daily maintenance work to achieve rapid location finding and precise positioning effects, reducing obstacles for emergency care work.

Villager spontaneous emergency forces and public security forces: Multi-party participation and transportation acceleration. When ambulance numbers are insufficient, besides applying for funding and resource assistance, village committees can also recruit villagers with private cars to voluntarily form emergency vehicle teams with unified management, while providing emergency skill training for drivers. Based on independent emergency care models, we can also try combining emergency care systems with multiple public security forces such as fire departments and police (39). For example, during emergencies, fire trucks from fire stations can be separately dispatched, and professionally trained “firefighter emergency care providers” with appropriate qualifications can provide pre-hospital emergency care services for patients (40). During ambulance hospital return trips, traffic police departments can view ambulance locations in real-time, open “green channels,” and allow ambulances unobstructed passage throughout their journey.

### 4.5 Reference significance of Chinese strategy for other countries

Globally, rural areas commonly face problems of slow emergency response, resource shortages, and insufficient system coordination in pre-hospital emergency care systems. Taking Japan as an example, research indicates that rural areas have insufficient numbers of emergency centers and uneven emergency personnel training levels, leading to extended emergency response times and low patient transportation efficiency (41). South Korea’s situation is also concerning. Due to insufficient doctor numbers, particularly in rural areas, emergency service coverage is limited (42). Additionally, emergency facilities and equipment in South Korea’s rural areas are relatively outdated and cannot meet rapid response and efficient treatment needs (43). Addressing these problems, our proposed “emergency central system—peripheral support system” coordination strategy can provide useful reference for these countries. For example, in rural areas of Japan and South Korea, regional emergency coordination centers can be established to integrate existing medical resources and emergency forces, improving emergency response speed and treatment capacity (44). Meanwhile, using information technology to achieve real-time emergency information sharing and transmission can ensure smooth emergency processes. Additionally, strengthening emergency personnel training to improve their professional skills and emergency response capabilities is also key to optimizing rural pre-hospital emergency care systems (45). By learning from our optimization strategies, these countries may be able to build more efficient, coordinated, and sustainable rural pre-hospital emergency care systems on their existing foundations, improving overall emergency treatment capabilities.

## 5 Conclusion

We focused specifically on the completeness and coordination aspects of China’s rural emergency care system construction. Based on our field research, we identified current problems in China’s rural emergency care, including low grassroots medical care levels, lack of coordination between the three-tier emergency care providers, and constraints from social and environmental factors. Furthermore, based on Bertalanffy’s general systems theory, we proposed a coordination strategy for improving China’s rural emergency care system consisting of an “emergency central system—emergency peripheral system” linkage approach. This strategy aims to provide new pathways for improving overall emergency capacity in rural areas while also offering Chinese experience for rural emergency care system construction in other countries around the world.

## Data Availability

The original contributions presented in the study are included in the article, further inquiries can be directed to the corresponding author.
